# Performance Improvement of the One-Dot Lateral Flow Immunoassay for Aflatoxin B1 by Using a Smartphone-Based Reading System

**DOI:** 10.3390/s130405109

**Published:** 2013-04-18

**Authors:** Sangdae Lee, Giyoung Kim, Jihea Moon

**Affiliations:** Post-Harvest & Food Engineering Division, Department of Agricultural Engineering, National Academy of Agricultural Sciences, Suwon 441707, Korea; E-Mails: sdlee96@gmail.com (S.L.); mmir95@gmail.com (J.M.)

**Keywords:** lateral flow immunoassay, aflatoxin B1, Smartphone, point-of-care

## Abstract

This study was conducted to develop a simple, rapid, and accurate lateral flow immunoassay (LFIA) detection method for point-of-care diagnosis. The one-dot LFIA for aflatoxin B1 (AFB1) was based on the modified competitive binding format using competition between AFB1 and colloidal gold-AFB1-BSA conjugate for antibody binding sites in the test zone. A Smartphone-based reading system consisting of a Samsung Galaxy S2 Smartphone, a LFIA reader, and a Smartphone application for the image acquisition and data analysis. The detection limit of one-dot LFIA for AFB1 is 5 μg/kg. This method provided semi-quantitative analysis of AFB1 samples in the range of 5 to 1,000 μg/kg. Using combination of the one-dot LFIA and the Smartphone-based reading system, it is possible to conduct a more fast and accurate point-of-care diagnosis.

## 1. Introduction

Aflatoxins are known as a toxic secondary metabolites produced by the some species of fungi of the genus *Aspergillus* [1]. The International Agency for Research on Cancer (IARC) has classified the aflatoxins B1 (AFB1), B2 (AFB2), G1 (AFG1), and G2 (AFG2) as Group-1 carcinogenic substances [2].AFB1 is one of the most potent hepato-carcinogens known, and the long-term chronic exposure to extremely low levels of AFB1 in food and feed is an important consideration for human and animal health. Consequently, maximum residue levels (MRL) of aflatoxins for human food and animal feed have been set by the European Union (EU). For maize and rice to be subjected to sorting or other physical treatment before human consumption or use as an ingredient in foodstuffs, AFB1 and total aflatoxin limits have been set at 5 μg/kg and 10 μg/kg [3].

To analyze AFB1 and aflatoxins, Several analytical methods including thin-layer chromatography (TLC), high-performance liquid chromatography (HPLC), liquid chromatography-mass spectroscopy (LC-MS), gas chromatography-mass spectroscopy (GS-MS), and enzyme-linked immunosorbent assay (ELISA) have been developed [4–7]. Because these methods are time-consuming, use expensive equipment, and require specialists, they are unsuitable for point-of-care diagnosis. The lateral flow immunoassay (LFIA) has gained increasing interest to overcome those problems. LFIA offers a low-cost, rapid and sensitive detection, user-friendly operation, easy storage, and point-of-care diagnosis. Recently, LFIA has been studied to detect mycotoxins such as aflatoxin B1, ochratoxin A, and fumonisin B1 [8–11]. Particularly, some LFIAs for quantitative or semi-quantitative analysis have been developed using a reading device. As there is an increasing need for high performing LIFA in the clinical, environmental, self-diagnosis, agriculture, and food safety areas [12–16], conventional LFIA having readout errors to the naked eye is up against some major problems such as poor quantitative discrimination, and low analytical sensitivity. To make the most out of LFIA's advantages such as moderate price, rapid point-of-care diagnosis, and the absence of need of expensive equipment and skilled personnel, LFIA readers measuring the optical densities of the LFIA detection area have been developed for point-of-care applications.

The objective of this study was to develop a more simple, rapid, and accurate LFIA detection method than conventional LFIA method for point-of-care diagnosis. The novel one-dot LFIA based on the competitive immunoassay was developed for AFB1 detection and a Smartphone-based reading system composed of a Smartphone, LFIA reader, and Smartphone application was fabricated for quantitative or semi-quantitative analysis. Using the Smartphone-based reading system, this study was conducted to improve the detection limit and sensitivity of the one-dot LFIA for AFB1 in maize and minimize the readout errors caused by a visual detection.

## 2. Materials and Methods

### 2.1. Materials

Aflatoxin B1 (AFB1), ochratoxin A (OTA), bovine serum albumin (BSA), AFB1-BSA conjugate, AFB1-polyclonal antibody (AFB1-pAb), borate buffer, Tween-20, sucrose, phosphate buffered saline (PBS), and other chemicals were purchased from Sigma-Aldrich Co. (St. Louis, MO, USA). Gold-in-a-Box kit with 40 nm gold nanoparticles was purchased from BioAssay Works (Ijamsville, MD, USA). For lateral flow immunoassay, sample pad (cellulose fiber, 17 × 300 mm), conjugation pad (glass fiber, 10 × 300 mm), nitrocellulose membrane (Hi Flow 240 membrane, 60 × 300 mm), and absorbent pad (cellulose fiber, 17 × 300 mm) were obtained from Merck Milipore (Billerica, MA, USA).

### 2.2. Preparation of LFIA

The one-dot LFIA for AFB1 was based on a LFIA method developed by Moon *et al*. [17]. The colloidal gold-AFB1-BSA and antibody concentrations were modified to achieve better sensitivity and detection limits. To determine the optimum condition of the colloidal gold-AFB1-BSA, the colloidal gold solutions were adjusted to pH 5.4–10.1 using the buffer solutions. 1 μL of AFB1-BSA solution was mixed with 100 μL of colloidal gold solution. After 30 min at room temperature, 10 μL of BSA blocking solution was added to the mixtures and the color of these solutions was observed. Because low or high pH conditions induce the gold nanoparticle aggregation, an insufficient amount of antigens are adsorbed on the surface of the gold nanoparticles. The aggregation can be visually detected because the red color of the colloidal gold solution is changed to blue-gray. The pH condition of the colloidal gold solution for the colloidal gold-AFB1-BSA conjugate was adjusted to pH 7.4.

The sample pad, nitrocellulose membrane, and conjugation pad were prepared, as previously described [17]. The sample pad was treated with 50 mM borate buffer, pH 7.4, containing 1% BSA and 0.05% Tween-20, and then dried overnight at 37 °C. The nitrocellulose membrane was blocked with PBS buffer. The conjugation pad was blocked with 50 mM borate buffer, pH 7.4, containing 10% sucrose, 2% BSA, and 0.05% Tween-20. And then, the membrane and conjugation pad were dried at 37 °C for 4 h. The test zone on the membrane was formed with 0.5 μL of AFB1-pAb (2.1 mg/mL in PBS), and allowed to dry at 37 °C for 3 h. 0.5 μL of colloidal gold-AFB1-BSA conjugate (colloidal gold:AFB1-BSA = 100:1) was applied to a conjugate pad and completely dried at 37 °C for 3 h. The sample pad, conjugate pad, nitrocellulose membrane, and absorption pad were assembled as the lateral flow strip. This strip was inserted into a plastic cassette, and these were stored at room temperature until use.

### 2.3. Sample Preparation

A maize sample was purchased from a retail store. The sample was ground using a household grinder and homogenized. Ground maize sample (25 g) was weighed and extracted with 125 mL of 5% methanol-PBS (v/v) using the mini shaker for 2 h. After centrifugation at 5,000 rpm, the clear supernatant was collected and analyzed. Different concentrations of AFB1 (0, 5, 10, 100, and 1,000 μg/kg) were added. Sample extract (100 μL) was added in the sample pad of the LFIA.

### 2.4. Interpretation of One-Dot LFIA

**Figure 1.**
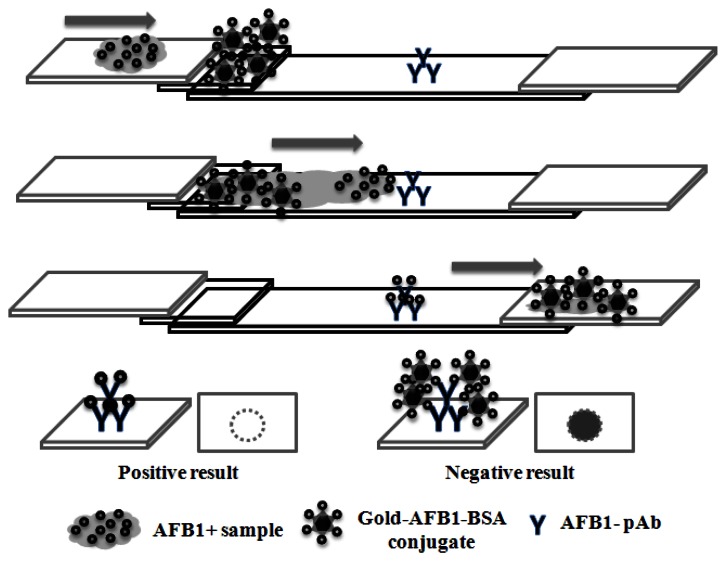
Schematic description of the one-dot LFIA for AFB1.

A schematic illustration of the one-dot LFIA is shown in Figure 1. The molecular weight of AFB1 is lower than that of the colloidal gold-AFB1-BSA conjugate, and the rate of AFB1 movement on the membrane is higher than that of the colloidal gold-AFB-BSA conjugate. AFB1-pAb is only able to combine with AFB1 or colloidal gold-AFB1-BSA conjugate. If there is AFB1 in the sample extract, AFB1 is combined faster with AFB1-pAb on the membrane than the colloidal gold-AFB1-BSA conjugate. At this point, the inhibition assay is completed and no red color occurs on the test zone. The positive result is judged by the absence of a one-dot on the membrane. However, if there is no AFB1 in the sample extract, the colloidal gold-AFB1-BSA conjugate combines with AFB1-pAb and the red color of test zone can be detected by visual inspection. The negative result is judged by the presence of one-dot on the membrane.

### 2.5. Instrumentation

**Figure 2.**
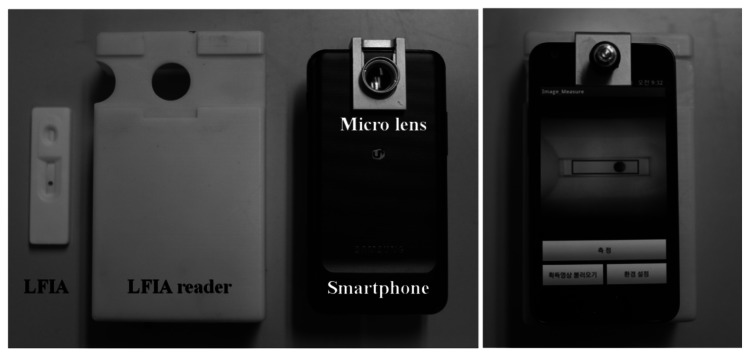
Smartphone-based reading system.

The Smartphone-based reading system consists of a Samsung Galaxy S2 Smartphone, LFIA reader, and Smartphone application, as shown in Figure 2. The LFIA reader is composed of the close-up lens with a 30 mm focal length, white LED light, lithium polymer battery, and main body. The Smartphone application for image acquisition and data analysis was developed on the Android platform.

**Figure 3.**
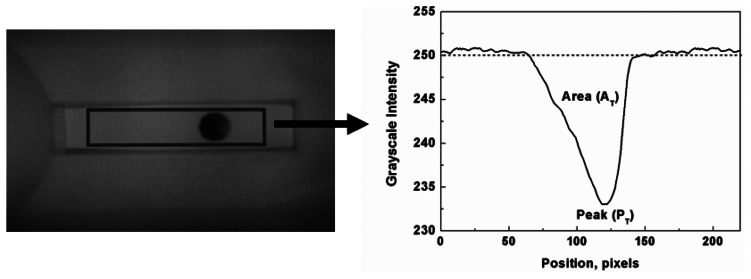
Typical photo image and intensity profile of detection area.

The analysis process of Smartphone-based reading system is as follows. The Smartphone camera was positioned on the close-up lens mounted in the top of LFIA reader. The white LED lights illuminated the detection area of LFIA, and the image of detection area was acquired using the Smartphone camera. The optical density of this image was measured by the Smartphone application, and the peak (P_T_) and area (A_T_) value of the test zone on the detection area were calculated as shown in Figure 3.

## 3. Results and Discussion

### 3.1. Detection Limit of LFIA for AFB1

**Figure 4.**
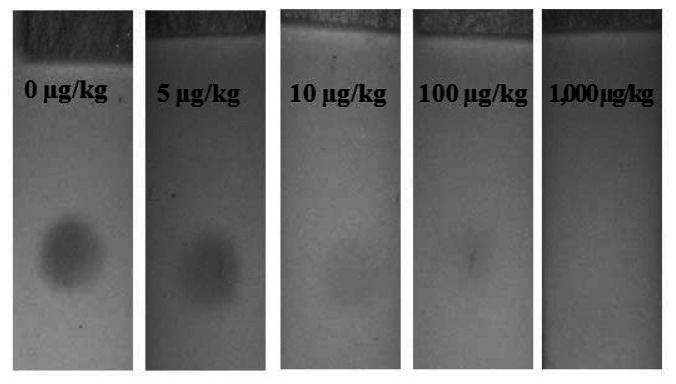
Detection limit of AFB1 with the one-dot LFIA.

The basic detection principle of LFIA is to allow visual detection of the presence or absence of aflatoxin B1 in sample extracts. The visual detection limit of LFIA is controlled by the amount of AFB1-pAb on the membrane and the colloidal gold-AFB1-BSA conjugate on the conjugation pad. The optimal manufacturing conditions of LFIA for AFB1 were determinate by the trial and error method. As the EU maximum residue limit (MRL) for AFB1 in maize and rice is established at 5 μg/kg, the visual detection limit of LFIA in this study was set at 5 μg/kg. The detection limit of LFIA for AFB1 is determined as the AFB1 concentration in the sample that causes the invisibility of the test zone. Each sample containing 0, 5, 10, 100, and 1,000 μg/kg concentration of AFB1 was assayed seven times using the LFIA and the results were judged by the visual inspection and Smartphone-based reading system. As shown in Figure 4, the detection limit of LFIA for AFB1 was 5 μg/kg. The color of test zone was clearly detected at 5 μg/kg of AFB1, but it disappeared at 10 μg/kg.

**Figure 5.**
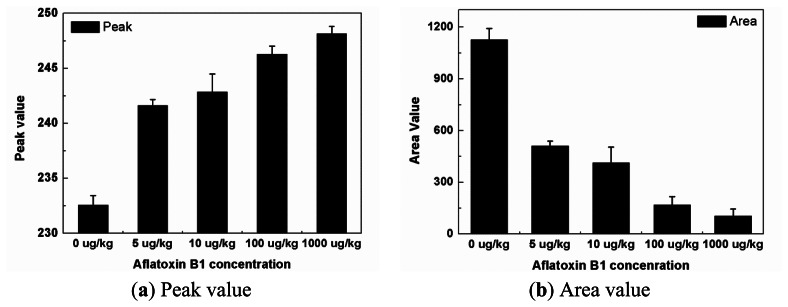
(**a**) Peak and (**b**) Area test results measured by the Smartphone-based reading system.

Figure 5(a,b) show the peak and area values measured by the Smartphone-based reading system. In Figure 5(a), the peak value is sharply increased at 5 μg/kg and then increased linearly with the log concentration. On the contrary, the area value is distinctly decreased in the same range and then decreased linearly with log concentrations in Figure 5(b). It is possible to conduct the linear regression analysis in between 5 and 1,000 μg/kg. The correlation coefficients of peak and area values are 0.969 and 0.948, respectively. In Figure 5, we can definitely separate the blank sample from the contaminated sample using the Smartphone-based reading system. The other advantage of this system is represented by the objectivity of the result, which does rely on the subjective interpretation of an operator.

### 3.2. Cross Reactivity of LFIA for AFB1

**Figure 6.**
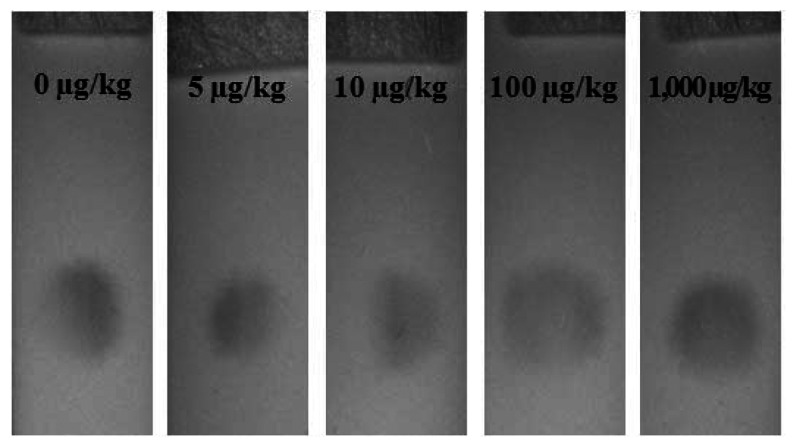
Cross-reactivity test results using OTA.

Ochratoxin A (OTA) is mainly produced by *Aspergillus* and also found in grain products. AFB1 and OTA are known hepatotoxins and potential carcinogens, therefore, the cross-reactivity of LFIA for AFB1 was tested to evaluate the reactivity between AFB1-pAb and OTA using 0, 5, 10, 100, and 1,000 μg/kg of OTA. In the blank sample and samples contaminated by OTA, the colors of test zones were uniformly detected, as shown in Figure 6. The results showed no cross-reactivity between AFB1-pAb and OTA. It is shown that the one-dot LFIA can be used for the detection of AFB1 in animal feeds and food.

## 4. Conclusions

A method for measuring AFB1 using the one-dot LFIA was developed and quantitative analysis was conducted using a Smartphone-based reading system. The one-dot LFIA was applied to modify the one-step membrane assay and LFIA. The Smartphone-based reading system can make the best use of the features of Smartphones such as extended battery life, increased processor speed, built-in megapixel camera, and the Android development platform. The detection limit of one-dot LFIA for AFB1 was 5 μg/kg. This result meets with the MRL established by the EU for AFB1 in maize and rice. Using the Smartphone-based reading system, we can separate the blank from contaminated samples on the base of peak and area values. A linear correlation for AFB1 was obtained in the range of 5 to 1,000 μg/kg. To overcome the need for well-trained experts and high performance equipment, the combination of the one-dot LFIA and the proposed Smartphone-based reading system can be useful for fast and accurate point-of-care diagnosis.
